# An Elementary Solution to a Duffing Equation

**DOI:** 10.1155/2022/2357258

**Published:** 2022-05-17

**Authors:** Alvaro H. Salas

**Affiliations:** Universidad Nacional de Colombia, Fizmako Research Group, Bogota, Colombia

## Abstract

In this work, we study the Duffing equation. Analytical solution for undamped and unforced case is provided for any given arbitrary initial conditions. An approximate analytical solution is given for the damped or trigonometrically forced Duffing equation for arbitrary initial conditions. The analytical solutions are expressed in terms of elementary trigonometric functions as well as in terms of the Jacobian elliptic functions. Examples are added to illustrate the obtained results. We also introduce new functions for approximating the Jacobian and Weierstrass elliptic functions in terms of the trigonometric functions sine and cosine. Results are high accurate.

## 1. Introduction

Many physical phenomena are modeled by nonlinear systems of ordinary differential equations. The Duffing equation is an externally forced and damped oscillator equation that exhibits a range of interesting dynamic behavior in its solutions. The Duffing oscillator is an important model of nonlinear and chaotic dynamics. It was introduced by Germanic engineer Duffing in 1918 [1]. The Duffing oscillator is described by the differential equation:

(1) $$\begin{array}{c} \ddot{x}+r\;\dot{x}+\omega_{0}^{2}x+\beta x^{3}=F\;\cos\;\omega t. \end{array}$$

It differs from the classical forced and damped harmonic oscillator only by the nonlinear term *βx*^3^, which changes the dynamics of the system drastically. Motivated by potential applications in physics, engineering, biology, and communication theory, the damped Duffing equation

(2) $$\begin{array}{c} \ddot{x}+r\;\dot{x}+\omega_{0}^{2}x+\beta x^{3}=0, \end{array}$$

is considered. Equation (2) is a ubiquitous model arising in many branches of physics and engineering, such as the study of oscillations of a rigid pendulum undergoing with moderately large amplitude motion [2, 3], vibrations of a buckled beam, and so on [3–5].

It has provided a useful paradigm for studying nonlinear oscillations and chaotic dynamical systems, dating back to the development of approximate analytical methods based on perturbative ideas [2], and continuing with the advent of fast numerical integration by the computer, to be used as an archetypal illustration of chaos [2, 5–7]. Various methods for studying the damped Duffing equation and the forced Duffing equation (1) in feedback control, strange attractor, stability, periodic solutions, and numerical simulations have been proposed, and a vast number of profound results have been established [2].

The Duffing equation has been studied extensively in the literature. However, only few works are devoted to the study of its analytical solutions not using perturbation methods [8, 9]. Our aim is to avoid using such perturbation methods. This study is organized as follows. In the first section, we give exact analytical solution for the undamped and unforced Duffing equation for any given arbitrary initial conditions. In the second section, we provide formulas for obtaining a good approximate analytical solution using a new ansatz. The problems are solved for any arbitrary initial conditions. Finally, in the last section, we give approximate analytical solution to (1) and we compare it with Runge–Kutta numerical solution. Other useful methods are the homotopy perturbation method (HPM) [10–17], the Lindstedt–Poincaré method, and the Krylov–Bogoliubov–Mitropolsky method. The importance of numerical solution of differential equations in different fields of science and engineering is given in [18, 19].

## 2. Undamped and Unforced Duffing Equation

This is the equation:

(3) $$\begin{array}{c} \ddot{x}+px+qx^{3}=0,\;x=x\left(t\right), \end{array}$$

and given the initial conditions,

(4) $$\begin{array}{c} x\left(0\right)=x_{0}\text{ and }x^{\prime }\left(0\right)={\dot{x}}_{0}\text{.} \end{array}$$

The general solution to equation (3) may be written in terms of any of the twelve Jacobian elliptic functions [20]. Let, for example,

(5) $$\begin{array}{c} x\left(t\right)=c_{1}\text{cn}\left(\sqrt{\omega }t+c_{2},m\right). \end{array}$$

Then,

(6) $$\begin{array}{c} \ddot{x}+px+qx^{3}=\left(c_{1}^{3}q-2c_{1}m\omega \right)cn^{3}+\left(2c_{1}m\omega +c_{1}p-c_{1}\omega \right)cn,\text{where }cn=cn\left(\sqrt{\omega }t+c_{2},m\right). \end{array}$$

Equating to zero, the coefficients of cn^*j*^ to zero gives an algebraic system whose solution is

(7) $$\begin{array}{c} \omega =\sqrt{p+qc_{1}^{2}}\text{ and }m=\frac{qc_{1}^{2}}{2\left(p+qc_{1}^{2}\right)}\text{.} \end{array}$$

Thus, the general solution to the Duffing equation is

(8) $$\begin{array}{c} x\left(t\right)=cn\left(\sqrt{p+qc_{1}^{2}}t+c_{2},\frac{qc_{1}^{2}}{2\left(p+qc_{1}^{2}\right)}\right). \end{array}$$

The values for the constants *c*_1_ and *c*_2_ are determined from the initial conditions.

**Definition 1 .**

The number $\Delta ={\left(p+qx_{0}^{2}\right)}^{2}+2q{\dot{x}}_{0}^{2}$ is called the discriminant for the Duffing equations (3) and (4).

We will distinguish three cases depending on the sign of the discriminant [20].

### 2.1. First Case: Δ > 0

The solution to the i.v.p. (3) and (4) is given by

(9) $$\begin{array}{c} x\left(t\right)=\sqrt{\frac{\sqrt{\Delta }-p}{q}}cn\left(\sqrt[{4}]{\Delta t}-\text{sign}\left({\dot{x}}_{0}\right)cn^{-1}\left(\sqrt{\frac{q}{\sqrt{\Delta }-p}}x_{0},\frac{1}{2}-\frac{p}{2\sqrt{\Delta }}\right),\frac{1}{2}-\frac{p}{2\sqrt{\Delta }}\right). \end{array}$$

Making use of the addition formula,

(10) $$\begin{array}{c} cn\left(x+y,m\right)=\frac{cn\left(x,m\right)cn\left(y,m\right)+sn\left(x,m\right)dn\left(x,m\right)sn\left(y,m\right)dn\left(y,m\right)}{1-msn\left(x,m\right)sn\left(y,m\right)}. \end{array}$$

The solution (9) may be expressed as

(11) $$\begin{array}{c} x\left(t\right)=\frac{x_{0}\text{cn}\left(\sqrt[{4}]{\Delta t}|m\right)+\left({\dot{x}}_{0}/\sqrt[{4}]{\Delta }\right)\text{sn}\left(\sqrt[{4}]{\Delta t}|m\right)\text{dn}\left(\sqrt[{4}]{\Delta t}|m\right)}{1+\left(\left(p+qx_{0}^{2}/2\sqrt{\Delta }\right)-\left(1/2\right)\right)\text{sn}{\left(\sqrt[{4}]{\Delta t}|m\right)}^{2}}, \end{array}$$

where

(12) $$\begin{array}{c} m=\frac{1}{2}\left(1-\frac{p}{\sqrt{\Delta }}\right). \end{array}$$

Solution (11) is a periodic solution with period

(13) $$\begin{array}{c} T=4\left|\frac{K\left(\left(1/2\right)\left(1-\left(p/\sqrt{\Delta }\right)\right)\right)}{\sqrt[{4}]{\Delta }}\right|. \end{array}$$

**Example 1 .**

Let us consider the i.v.p.

(14) $$\begin{array}{c} \left\{\begin{array}{c} x^{\prime \prime }\left(t\right)+x\left(t\right)+x^{3}\left(t\right)=0,\\ x\left(0\right)=1\&x^{\prime }\left(0\right)=-1. \end{array}\right. \end{array}$$

Using formula (9), the exact solution to (14) is given by

(15) $$\begin{array}{c} x\left(t\right)=\sqrt{\sqrt{6}-1}cn\left(\sqrt[{4}]{6t}-cn^{-1}\left(\frac{1}{\sqrt{-1+\sqrt{6}}},\frac{1}{2}-\frac{1}{2\sqrt{6}}\right)|\frac{1}{2}-\frac{1}{2\sqrt{6}}\right). \end{array}$$

According to the relations (11) and (12), the exact solution to the i.v.p. (14) may also be written as

(16) $$\begin{array}{c} x\left(t\right)=\frac{cn\left(\sqrt[{4}]{6t}|\left(1/2\right)|\left(1-\left(1/\sqrt{6}\right)\right)\right)+\sqrt[{4}]{6\;dn}\left(\sqrt[{4}]{6t}|\left(1/2\right)|\left(1-\left(1/\sqrt{6}\right)\right)\right)sn\left(\sqrt[{4}]{6t}|\left(1/2\right)|\left(1-\left(1/\sqrt{6}\right)\right)\right)}{1+\left(\left(2-\sqrt{6}\right)/2\sqrt{6}\right)sn{\left(\sqrt[{4}]{6t}|\left(1/2\right)|\left(1-\left(1/\sqrt{6}\right)\right)\right)}^{2}}. \end{array}$$

The period is given by

(17) $$\begin{array}{c} T=\frac{2\sqrt[{4}]{8}}{\sqrt[{4}]{3}}K\left(\frac{1}{2}\left(1-\frac{1}{\sqrt{6}}\right)\right)\approx 4.37417. \end{array}$$

**Figure 1:**
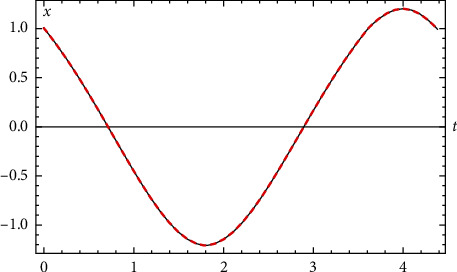
Comparison between the exact solution and the numerical solution for Example 1.

In Figure 1, the comparison between the exact analytical solution (??) and the approximate numerical RK4 solution is presented. Full compatibility between the two analytical and numerical solutions is observed.

### 2.2. Second Case: Δ < 0

In this case, *q* < 0. Define

(18) $$\begin{array}{c} \delta =-\frac{2px_{0}^{2}+qx_{0}^{4}+2{\dot{x}}_{0}^{2}}{q}. \end{array}$$

Observe that

(19) $$\begin{array}{c} \delta =\frac{p^{2}-\Delta }{q^{2}}>0. \end{array}$$

Let

(20) $$\begin{array}{c} x\left(t\right)=c-\frac{2c}{1+y\left(t\right)}, \end{array}$$

where *y*=*y*(*t*) is a solution to Duffing equation

(21) $$\begin{array}{c} y^{\prime \prime }\left(t\right)+ay\left(t\right)+by^{3}\left(t\right) \end{array}$$

with initial conditions

(22) $$\begin{array}{c} y\left(0\right)=y_{0}=\frac{2c{\dot{x}}_{0}}{{\left(c-x_{0}\right)}^{2}}\text{ and }y\prime \left(0\right)={\dot{y}}_{0}=\frac{c+x_{0}}{c-x_{0}}. \end{array}$$

Inserting ansatz (21) into the ode *x*′′(*t*)+*px*(*t*)+*qx*^3^(*t*)=0 and taking into account the relation,

(23) $$\begin{array}{c} y^{\prime }{\left(t\right)}^{2}={\dot{y}}_{0}+ay_{0}^{2}+\frac{b}{2}y_{0}^{4}-ay^{2}\left(t\right)-\frac{b}{2}y^{4}\left(t\right), \end{array}$$

we get

(24) $$\begin{array}{c} \frac{cy\left(t\right)-c-x_{0}y\left(t\right)-x_{0}}{4{\left(c-x_{0}\right)}^{4}{\left(y\left(t\right)+1\right)}^{4}}\left(\ddot{x}+px+qx^{3}\right)=\left(\begin{array}{c} 8ac^{5}-8ac^{4}x_{0}-8ac^{3}x_{0}^{2}+8ac^{2}x_{0}^{3}+4bc^{5}+12bc^{4}x_{0}+12bc^{3}x_{0}^{2}+4bc^{2}x_{0}^{3}+c^{7}q-5c^{6}qx_{0}+2c^{5}p\\ +11c^{5}qx_{0}^{2}-10c^{4}px_{0}-15c^{4}qx_{0}^{3}+20c^{3}px_{0}^{2}+15c^{3}qx_{0}^{4}+30c^{3}{\dot{x}}_{0}^{2}-20c^{2}px_{0}^{3}-11c^{2}qx_{0}^{5}\\ -22c^{2}x_{0}{\dot{x}}_{0}^{2}+10cpx_{0}^{4}+5cqx_{0}^{6}+10cx_{0}^{2}{\dot{x}}_{0}^{2}-2px_{0}^{5}-qx_{0}^{7}-2x_{0}^{3}{\dot{x}}_{0}^{2} \end{array}\right)\\ +\left(c-x_{0}\right)\left(\begin{array}{c} 8ac^{4}-16ac^{3}x_{0}+8ac^{2}x_{0}^{2}+4bc^{4}+8bc^{3}x_{0}+4bc^{2}x_{0}^{2}-3c^{6}q+10c^{5}qx_{0}+2c^{4}p-11c^{4}qx_{0}^{2}-\\ 12c^{3}px_{0}+24c^{2}px_{0}^{2}+11c^{2}qx_{0}^{4}+22c^{2}{\dot{x}}_{0}^{2}-20cpx_{0}^{3}-10cqx_{0}^{5}-20cx_{0}{\dot{x}}_{0}^{2}+6px_{0}^{4}+3qx_{0}^{6}+6x_{0}^{2}{\dot{x}}_{0}^{2} \end{array}\right)y\left(t\right)\\ +{\left(c-x_{0}\right)}^{2}\left(4bc^{3}+4bc^{2}x_{0}+3c^{5}q-5c^{4}qx_{0}-2c^{3}p-2c^{2}px_{0}+10cpx_{0}^{2}+5cqx_{0}^{4}+10c{\dot{x}}_{0}^{2}-6px_{0}^{3}-3qx_{0}^{5}-6x_{0}{\dot{x}}_{0}^{2}\right)y^{2}\left(t\right)+{\left(c-x_{0}\right)}^{3}\left(4bc^{2}-qc^{4}-2c^{2}p+2px_{0}^{2}+qx_{0}^{4}+2{\dot{x}}_{0}^{2}\right)y^{3}\left(t\right). \end{array}$$

Equating to zero, the coefficients of *y*^*j*^(*t*) give an algebraic system. A solution to this system is

(25) $$\begin{array}{c} a=\frac{1}{2}\left(-p-3\sqrt{\delta }q\right),b=\frac{1}{2}\left(p-\sqrt{\delta }q\right),c=\sqrt[{4}]{\delta }. \end{array}$$

Observe that the Duffing equations (21) and (22) have a positive discriminant given by

(26) $$\begin{array}{c} {\left(a+by_{0}^{2}\right)}^{2}+2b\;{\dot{y}}_{0}^{2}=\frac{\delta {\left(c-x_{0}\right)}^{4}\left(2c^{4}{\dot{x}}_{0}^{2}+\delta {\left(c^{2}+x_{0}^{2}\right)}^{2}\right)}{4c^{8}x_{0}^{2}}. \end{array}$$

Then, the problem reduces to the first case.

**Example 2 .**

Let us assume the following i.v.p.:

(27) $$\begin{array}{c} x^{\prime \prime }\left(t\right)+2x\left(t\right)-x^{3}\left(t\right)=0,x\left(0\right)=-1\text{ and }x^{\prime }\left(0\right)=1. \end{array}$$

The solution of i.v.p. (27) according to the relation (??) reads

(28) $$\begin{array}{c} x\left(t\right)=1.49535-\frac{2.9907}{1+\left(\left(0.198509cn\left(2.08627t|1.00018\right)-0.230219\;dn\left(2.08627t|1.00018\right)sn\left(2.08627t|1.00018\right)\right)/1-1.00072sn{\left(2.08627t|1.00018\right)}^{2}\right)}. \end{array}$$

The period of solution (28) is given by

(29) $$\begin{array}{c} T=\frac{4K\left(1/m\right)}{w\sqrt{m}}=10.9034\left(\text{for }m=1.00018\text{ and }w=2.08627\right). \end{array}$$

**Figure 2:**
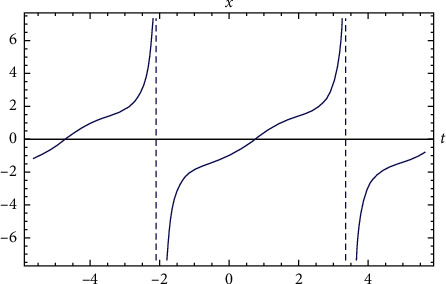
Comparison between the exact solution and the numerical solution for Example 2.

Comparison between the exact solution and numerical solution is shown in Figure 2.

### 2.3. Third Case: Δ=0 and *p* ≠ 0

If the discriminant vanishes (Δ=0), then *q* < 0, and the only solution to problem (??) with

(30) $$\begin{array}{c} x^{\prime }{\left(0\right)}^{2}={\dot{x}}_{0}^{2}=\frac{{\left(p+qy_{0}^{2}\right)}^{2}}{-2q} \end{array}$$

reads

(31) $$\begin{array}{c} x\left(t\right)=\sqrt{-\frac{p}{q}}\tanh\left[\sqrt{\frac{p}{2}}t\pm \;\tanh^{-1}\left(x_{0}\sqrt{-\frac{q}{p}}\right)\right], \end{array}$$

which may be verified by direct computation.

### 2.4. Fourth Case: Δ=0 and *p*=0

The solution is given by

(32) $$\begin{array}{c} x\left(t\right)=\frac{2x_{0}}{2+\sqrt{-2q}x_{0}\;t}. \end{array}$$

**Remark 1 .**

The solution to the i.v.p.

(33) $$\begin{array}{c} \ddot{x}+px+qx^{3}=0\text{, }x\left(0\right)=x_{0}\text{ and }x^{\prime }\left(0\right)=0, \end{array}$$

is

(34) $$\begin{array}{c} x\left(t\right)=x_{0}cn\left(\sqrt{p+qx_{0}^{2}},\frac{qx_{0}^{2}}{2\left(p+qx_{0}^{2}\right)}\right). \end{array}$$

**Remark 2 .**

Let $p+\sqrt{p^{2}+2q{\dot{x}}_{0}^{2}}>0$. Then, the solution to the i.v.p.

(35) $$\begin{array}{c} \ddot{x}+px+qx^{3}=0,\;x\left(0\right)=0\text{ and }x\prime \left(0\right)={\dot{x}}_{0}, \end{array}$$

is

(36) $$\begin{array}{c} x\left(t\right)=\frac{\sqrt{2}{\dot{x}}_{0}}{\sqrt{\sqrt{p^{2}+2q{\dot{x}}_{0}^{2}}+p}}sn\left(\sqrt{\frac{p+\sqrt{p^{2}+2q{\dot{x}}_{0}^{2}}}{2}}t,-\frac{p^{2}+q{\dot{x}}_{0}^{2}-\sqrt{p^{2}+2q{\dot{x}}_{0}^{2}}p}{q{\dot{x}}_{0}^{2}}\right). \end{array}$$

**Remark 3 .**

Using the identity

(37) $$\begin{array}{c} cn\left(\sqrt{\omega }t,m\right)=1-\frac{6}{\left(4m+1\right)\left(1+12/\left(4m+1\right)\omega ℘\left(t;1/12\left(16m^{2}-16m+1\right)\omega^{2},1/216\left(2m-1\right)\left(32m^{2}-32m-1\right)\omega^{3}\right)\right)}, \end{array}$$

the solution to the Duffing equations (3) and (4) may be written in terms of the Weierstrass elliptic function *℘*. More precisely, if Δ > 0, then

(38) $$\begin{array}{c} x\left(t\right)=A-\frac{A\left(2+\left(4p/3A^{2}q+p\right)\right)}{1+\left(12/3A^{2}q+p\right)℘\left(t+t_{0};\left(1/12\right)\left(-3q^{2}A^{4}-6pqA^{2}+p^{2}\right),\left(1/216\right)p\left(9q^{2}A^{4}+18pqA^{2}+p^{2}\right)\right)}, \end{array}$$

where

(39) $$\begin{array}{c} t_{0}={℘}^{-1}\left(\frac{3A^{3}q+3A^{2}qx_{0}+5Ap+px_{0}}{12\left(A-x_{0}\right)};\frac{1}{12}\left(-3A^{4}q^{2}-6A^{2}pq+p^{2}\right),\frac{1}{216}p\left(9A^{4}q^{2}+18A^{2}pq+p^{2}\right)\right), \end{array}$$

(40) $$\begin{array}{c} A=\sqrt{\frac{-p\pm \sqrt{{\left(p+qx_{0}^{2}\right)}^{2}+2q{\dot{x}}_{0}^{2}}}{q}}=\pm \sqrt{\frac{-p\pm \sqrt{\Delta }}{q}}. \end{array}$$

The solution (38) is periodic with period

(41) $$\begin{array}{c} T=2\int_{\rho }^{+\infty }\frac{dx}{\sqrt{4x^{3}-g_{2}x-g_{3}}}, \end{array}$$

where *ρ* is the greatest real root to the cubic 4*x*^3^ − *g*_2_*x* − *g*_3_=0 and

(42) $$\begin{array}{c} g_{2}=\frac{1}{12}\left(-3q^{2}A^{4}-6pqA^{2}+p^{2}\right)\text{ and }g_{3}=\frac{1}{216}p\left(9A^{4}q^{2}+18A^{2}pq+p^{2}\right). \end{array}$$

On the other hand,

(43) $$\begin{array}{c} ℘\left(t;g_{2},g_{3}\right)=\frac{\sqrt{3g_{2}}}{\sqrt{16m^{2}-16m+1}\left(1-\;cn\left(\sqrt{2}\sqrt[{4}]{3g_{2}/16m^{2}-16m+1}t,m\right)\right)}-\frac{\sqrt{g_{2}}\left(4m+1\right)}{2\sqrt{48m^{2}-48m+3}}, \end{array}$$

where *m* is a root to the sextic

(44) $$\begin{array}{c} 4096\left(g_{2}^{3}-27g_{3}^{2}\right)z^{6}-12288\left(g_{2}^{3}-27g_{3}^{2}\right)z^{5}+13056\left(g_{2}^{3}-27g_{3}^{2}\right)z^{4}-5632\left(g_{2}^{3}-27g_{3}^{2}\right)z^{3}+12\left(59g_{2}^{3}-1836g_{3}^{2}\right)z^{2}+12\left(5g_{2}^{3}+108g_{3}^{2}\right)z+g_{2}^{3}-27g_{3}^{2}=0. \end{array}$$

Thus,

(45) $$\begin{array}{c} \int_{\rho }^{+\infty }\frac{dx}{\sqrt{4x^{3}-g_{2}x-g_{3}}}=\sqrt{2}\sqrt[{4}]{\frac{16m^{2}-16m+1}{3g_{2}}}K\left(m\right)\text{ for }m<1. \end{array}$$

## 3. Approximate Analytical Solution Using Elementary Functions

We define the generalized sine and cosine functions as follows:

(46) $$\begin{array}{c} \sin_{m}\left(t\right)=\frac{\sin\left(\sqrt{1+\kappa }t\right)}{\sqrt{1+\kappa \;\cos^{2}\left(\sqrt{1+\kappa }t\right)}}, \end{array}$$

(47) $$\begin{array}{c} \cos_{m}\left(t\right)=\frac{\sqrt{1+\kappa }\cos\left(\sqrt{1+\kappa }t\right)}{\sqrt{1+\kappa \;\cos^{2}\left(\sqrt{1+\kappa }t\right)}}, \end{array}$$

(48) $$\begin{array}{c} \kappa =\frac{1}{14}\left(m-12+\sqrt{144-144m+m^{2}}\right). \end{array}$$

These functions are good approximations to the Jacobian elliptic functions sn and cn for −1 ≤ *m* ≤ 1/2. For example, let

(49) $$\begin{array}{c} T=2K\left(m\right)\approx \frac{\pi }{\sqrt{1+\kappa }}. \end{array}$$

Then,

(50) $$\begin{array}{c} {\text{Max}}_{-T\leq t\leq T,-1\leq m\leq \left(1/2\right)}\left|\text{sn}\left(t,m\right)-\;\sin_{\text{m}}\left(\text{t}\right)\right|=0.00290582, \end{array}$$

(51) $$\begin{array}{c} {\text{Max}}_{-T\leq t\leq T,-1\leq m\leq 1/2}\left|cn\left(t,m\right)-\;\cos_{m}\left(t\right)\right|=0.00541969. \end{array}$$

Table 1 provides the errors for different values of *m*.

More accurate approximations are obtained by letting

(52) $$\begin{array}{c} \sin_{m}\left(t\right)=\frac{\sin\left(\sqrt{1+\kappa }t\right)}{\sqrt{1+\kappa \;\cos^{2}\left(\sqrt{1+\kappa }t\right)+\mu \;\cos^{4}\left(\sqrt{1+\kappa }t\right)}}, \end{array}$$

(53) $$\begin{array}{c} \cos_{m}\left(t\right)=\frac{\sqrt{1+\kappa +\mu }\cos\left(\sqrt{1+\kappa }t\right)}{\sqrt{1+\kappa \;\cos^{2}\left(\sqrt{1+\kappa }t\right)+\mu \;\cos^{4}\left(\sqrt{1+\kappa }t\right)}}, \end{array}$$

(54) $$\begin{array}{c} \sin_{m}\left(t\right)=\frac{\sin\left(t\sqrt{-\kappa -\mu -1}/\sqrt{\mu -1}\right)\sqrt{1-\mu \;\cos^{2}\left(t\sqrt{-\kappa -\mu -1}/\sqrt{\mu -1}\right)}}{\sqrt{\mu \;\cos^{4}\left(t\sqrt{-\kappa -\mu -1}/\sqrt{\mu -1}\right)+\kappa \;\cos^{2}\left(t\sqrt{-\kappa -\mu -1}/\sqrt{\mu -1}\right)+1}}. \end{array}$$

(55) $$\begin{array}{c} \kappa =\frac{\left(\sqrt{\begin{array}{c} \left(5184m^{2}-14256m+15633\right)\mu^{4}+\left(-24192m^{2}+92016m-89856\right)\mu^{3}+\\ \left(42048m^{2}-211392m+193536\right)\mu^{2}+\left(-32256m^{2}+207360m-184320\right)\mu \\ +9216m^{2}-73728m+65536 \end{array}}\right)+\left(-72m-9\right)\mu^{2}+\left(168m-960\right)\mu -96m-256}{48\left(3\mu +10\right)} \end{array}$$

(56) $$\begin{array}{c} \mu =\frac{4m^{2}\left(1061m^{4}-7768m^{3}+24128m^{2}-32768m+16384\right)}{10521m^{6}-136752m^{5}+786336m^{4}-2345984m^{3}+3792896m^{2}-3145728m+1048576}. \end{array}$$

For these new approximations, we will have

(57) $$\begin{array}{c} {\text{Max}}_{-T\leq t\leq T,-1\leq m\leq 0.9}\left|sn\left(t,m\right)-\;\sin_{m}\left(t\right)\right|=0.0607706, \end{array}$$

(58) $$\begin{array}{c} {\text{Max}}_{-T\leq t\leq T,-1\leq m\leq 0.9}\left|cn\left(t,m\right)-\;\cos_{m}\left(t\right)\right|=0.030797. \end{array}$$

Table 2 provides the errors for different values of *m*.

From Tables 1 and 2, it is seen that for the values 0.8 < *m* < 1, the approximations (46) and (47) are better than (53) and (54). Thus, we have

(59) $$\begin{array}{c} sn\left(t,m\right)\approx \;\sin_{m}\left(t\right),cn\left(t,m\right)\approx \;\cos_{m}\left(t\right)\text{ and }dn\left(t,m\right)\approx \sqrt{1-msn^{2}\left(t,m\right)}=dn_{m}\left(t\right)\text{ for }-1\leq m<1. \end{array}$$

We may write approximate elementary solution to Duffing equations (3) and (4) as follows:

(60) $$\begin{array}{c} x\left(t\right)=\frac{x_{0}\cos_{m}\left(\sqrt[{4}]{\Delta t}\right)+{\dot{x}}_{0}/\sqrt[{4}]{\Delta }\sin_{m}\left(\sqrt[{4}]{\Delta t}\right){\text{dn}}_{m}\left(\sqrt[{4}]{\Delta t}\right)}{1+\left(\left(p+qx_{0}^{2}/2\sqrt{\Delta }\right)-\left(1/2\right)\right)\sin_{m}{\left(\sqrt[{4}]{\Delta t}\right)}^{2}}, \end{array}$$

where

(61) $$\begin{array}{c} m=\frac{1}{2}\left(1-\frac{p}{\sqrt{\Delta }}\right)\;,\;p>0,\;\Delta ={\left(p+qx_{0}^{2}\right)}^{2}+2q{\dot{x}}_{0}^{2}>0. \end{array}$$

(62) $$\begin{array}{c} x\left(t\right)=A-\frac{2A}{1+B\left(b_{0}\cos_{m}\left(\sqrt{\omega }t\right)+b_{1}\sin_{m}\left(\sqrt{\omega }t\right)dn_{m}\left(\sqrt{\omega }t\right)/1+b_{2}\sin_{m}{\left(\sqrt{\omega }t\right)}^{2}\right)},\;\Delta <0. \end{array}$$

The values for the constants in (62) are the same as in (??).

**Remark 4 .**

In the case when |*m*| > 1, we use the approximations:

(63) $$\begin{array}{c} cn\left(t,m\right)\approx dn_{1/m}\left(\sqrt{m}t\right), \end{array}$$

(64) $$\begin{array}{c} sn\left(t,m\right)\approx \sqrt{\frac{1}{m}}\sin_{1/m}\left(\sqrt{m}t\right), \end{array}$$

(65) $$\begin{array}{c} dn\left(t,m\right)\approx \;\cos_{1/m}\left(\sqrt{m}t\right). \end{array}$$

Then, formula (60) takes the following form when *p* < 0 and *m* > 1:

(66) $$\begin{array}{c} x\left(t\right)=\frac{x_{0}{\text{dn}}_{1/m}\left(\sqrt[{4}]{\Delta }\sqrt{m}t\right)+{\dot{x}}_{0}/\sqrt[{4}]{\Delta }\sqrt{1/m}\sin_{\left(1/m\right)}\left(\sqrt{m}\sqrt[{4}]{\Delta t}\right){\text{cos}}_{\left(1/m\right)}\left(\sqrt[{4}]{\Delta }\sqrt{m}t\right)}{1+\left(1/m\right)\left(\left(p+qx_{0}^{2}/2\sqrt{\Delta }\right)-\left(1/2\right)\right)\sin_{1/m}^{2}\left(\sqrt[{4}]{\Delta }\sqrt{m}t\right)}\text{, }\Delta >0. \end{array}$$

Similar formula for (62) when Δ < 0.

In the case when 0.9 < *m* ≤ 1.1, we may use the following approximations:

(67) $$\begin{array}{c} \left\{\begin{array}{c} cn\left(t,m\right)\approx \frac{1}{8}\left(m-1\right)\left(\sinh\left(2t\right)-2t\right)\tanh\left(t\right)\sec\;h\left(t\right)+\sec\;h\left(t\right),\\ sn\approx \frac{1}{4}\left(\left(m-1\right)t\;\sec\;h^{2}\left(t\right)-\left(m-5\right)\tanh\left(t\right)\right),\\ dn\;\approx \;\sec\;h\left(t\right)-\frac{1}{4}\left(m-1\right)\tanh\left(t\right)\left(\sinh\left(t\right)+t\;\sec\;h\left(t\right)\right). \end{array}\right. \end{array}$$

**Example 3 .**

Let us return to Example 1.

(68) $$\begin{array}{c} \left\{\begin{array}{c} x^{\prime \prime }\left(t\right)+x\left(t\right)+x^{3}\left(t\right)=0,\\ x\left(0\right)=1\;\&x^{\prime }\left(0\right)=-1.\; \end{array}\right. \end{array}$$

The approximate elementary analytical solution is

(69) $$\begin{array}{c} x_{app}\left(t\right)=\frac{\left(\left(3.4319\;\cos\left(1.43552t\right)\right)/\sqrt{14.-2.22208\;\cos^{2}\left(1.43552t\right)}\right)-\left(0.638943\;\sin\left(1.43552t\right)\sqrt{1+\left(4.14226\;\sin^{2}\left(1.43552t\right)/2.22208\;\cos^{2}\left(1.43552t\right)-14.\right)}/\sqrt{1.-0.15872\;\cos^{2}\left(1.43552t\right)}\right)}{1-\left(0.0917517\;\sin^{2}\left(1.43552t\right)/1.-0.15872\;\cos^{2}\left(1.43552t\right)\right)}. \end{array}$$

The exact period is given by *T*=4.37417. The approximate period is that of (59), and it is given by

(70) $$\begin{array}{c} T_{\text{app}}=\frac{2\pi }{1.4355163606}=4.37695. \end{array}$$

This value differs from the exact value by 0.00278457. The error of the approximate solution comparted with exact solution is

(71) $$\begin{array}{c} \max_{-T/2\leq t\leq T/2}\left|x_{\text{app}}\left(t\right)-x_{\text{exact}}\left(t\right)\right|=0.001847. \end{array}$$

**Figure 3:**
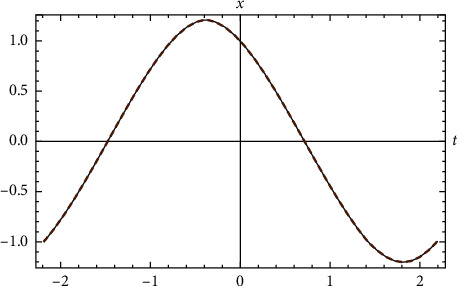
Comparison between the exact solution and the approximate analytical solution for Example 3.

Comparison between the exact solution and the approximate analytical solution is shown in Figure 3.

**Example 4 .**

**Figure 4:**
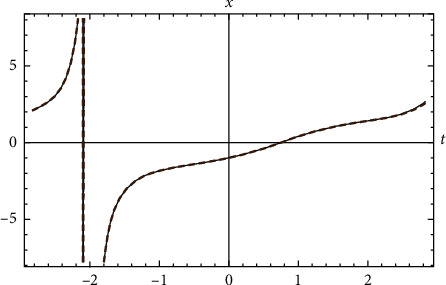
Comparison between the exact solution and the approximate analytical solution for Example 4.

Let us return to Example 2. Let (Figure 4)

(72) $$\begin{array}{c} x^{\prime \prime }\left(t\right)+2x\left(t\right)-x^{3}\left(t\right)=0,\;x\left(0\right)=-1\text{ and }x\left(0\right)=1. \end{array}$$

**Remark 5 .**

From (47)–(44) or (53)–(44), we obtain the following approximate expression for the Weierstrass elliptic function by means of the cosine function:

(73) $$\begin{array}{c} ℘\left(t;g_{2},g_{3}\right)=\frac{\sqrt{3g_{2}}}{\sqrt{16m^{2}-16m+1}\left(1-{\text{ cos}}_{m}\left(\sqrt{2}\sqrt[{4}]{3g_{2}/16m^{2}-16m+1}t\right)\right)}-\frac{\sqrt{g_{2}}\left(4m+1\right)}{2\sqrt{48m^{2}-48m+3}}, \end{array}$$

where *m* is a root to the sextic (44).

**Example 5 .**

Let *g*_2_=2 and *g*_3_=1. For this choice, *m*=0.0119056. We have

(74) $$\begin{array}{c} ℘\left(t;2,1\right)\approx \frac{2.71867}{1-\left(3.73048\;\cos\left(2.32484t\right)/\sqrt{14.-0.0835474\;\cos^{2}\left(2.32484t\right)}\right)}-0.474691. \end{array}$$

**Figure 5:**
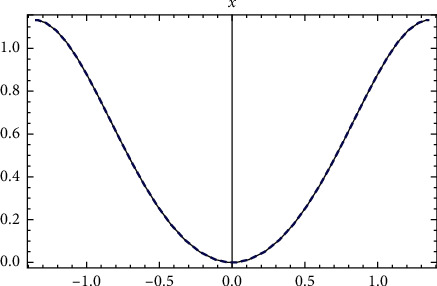
Comparison between the approximate analytical solution and the numerical solution for Example 5.

The period is *T*=2.70262, and the error on the interval −(*T*/2) ≤ *t* ≤ (*T*/2) in the sup norm is *E*=8.52 × 10^−7^. Comparison between the approximate analytical solution and the numerical solution is shown in Figure 5. The reciprocals of the two functions are plotted on the interval −(*T*/2) ≤ *t* ≤ (*T*/2).

## 4. Analytical Solution to a Generalized Duffing Equation

Let us consider the i.v.p. [21]:

(75) $$\begin{array}{c} \ddot{u}+\alpha u+\beta u^{3}=F, \end{array}$$

given that

(76) $$\begin{array}{c} u\left(0\right)=u_{0}\text{ and }u\prime \left(0\right)={\dot{u}}_{0}. \end{array}$$

We will say that (75) is a constantly forced Duffing equation. When *F*=0, that becomes an undamped and unforced Duffing equation, and we already know how to solve it for arbitrary initial conditions. Let

(77) $$\begin{array}{c} x\left(t\right)=\lambda +\frac{\mu }{1+℘\left(\rho \;t+t_{0};g_{2},g_{3}\right)}, \end{array}$$

where *λ*, *μ*, *w*, *g*_2_, *g*_3_, and *t*_0_ are some constants to be determined. Plugging ansatz (77) into (75) gives

(78) $$\begin{array}{c} \ddot{u}+\alpha u+\beta u^{3}-F=\frac{1}{2{\left(1+℘\right)}^{3}}\left(\begin{array}{c} -2\left(F-\alpha \lambda -\beta \lambda^{3}-2\mu \rho^{2}\right)-2\left(3F-3\alpha \lambda -\alpha \mu -3\beta \lambda^{3}-3\beta \lambda^{2}\;\mu +6\mu \rho^{2}\right)℘+\\ \left(-6F-3g_{2}\mu \rho^{2}+6\alpha \lambda +4\alpha \mu +6\beta \lambda^{3}+12\beta \lambda^{2}\mu +6\beta \lambda \mu^{2}\right){℘}^{2}+\\ \left(-2F+g_{2}\mu \rho^{2}-4g_{3}\mu \rho^{2}+2\alpha \lambda +2\alpha \mu +2\beta \lambda^{3}+6\beta \lambda^{2}\mu +6\beta \lambda \mu^{2}+2\beta \mu^{3}\right){℘}^{3} \end{array}\right), \end{array}$$

where *℘*=*℘*(*ρ* *t*+*t*_0_; *g*_2_, *g*_3_). Equating to zero, the coefficients of *℘*^*j*^ (*j*=0,1,2,3) in the right-hand side of (78) gives an algebraic system. A nontrivial solution to this system is

(79) $$\begin{array}{c} \left\{\begin{array}{c} \mu =\frac{6\left(F-\lambda \left(\alpha +\beta \lambda^{2}\right)\right)}{\alpha +3\beta \lambda^{2}},\;\rho =\frac{1}{2}\sqrt{\frac{\alpha }{3}+\beta \lambda^{2}},\\ g_{2}=12-\frac{144\beta \lambda \left(-F+\alpha \lambda +\beta \lambda^{3}\right)}{{\left(\alpha +3\beta \lambda^{2}\right)}^{2}},\;g_{3}=\frac{8\left(27F^{2}\beta +9\alpha \beta \lambda \left(\beta \lambda^{3}-4F\right)+\alpha^{3}+18\alpha^{2}\beta \lambda^{2}\right)}{{\left(\alpha +3\beta \lambda^{2}\right)}^{3}}. \end{array}\right. \end{array}$$

Now, to find the values of *t*_0_ and *λ*, we make use of the addition formula:

(80) $$\begin{array}{c} ℘\left(w+z;g_{2},g_{3}\right)=\frac{1}{4}{\left(\frac{{℘}^{\prime }\left(w;g_{2},g_{3}\right)-{℘}^{\prime }\left(z;g_{2},g_{3}\right)}{℘\left(w;g_{2},g_{3}\right)-℘\left(z;g_{2},g_{3}\right)}\right)}^{2}-℘\left(w;g_{2},g_{3}\right)-℘\left(z;g_{2},g_{3}\right). \end{array}$$

We then find that

(81) $$\begin{array}{c} t_{0}={℘}^{-1}\left(\frac{u_{0}-\lambda -\mu }{\lambda -u_{0}};g_{2},g_{3}\right). \end{array}$$

The number *λ* must be a solution to the quartic

(82) $$\begin{array}{c} 4Fu_{0}-\beta u_{0}^{4}-2u_{0}^{2}\alpha -2{\dot{u}}_{0}^{2}-4F\lambda +2\alpha \lambda^{2}+\beta \lambda^{4}=0. \end{array}$$

Using (75), we also may obtain an approximate analytical solution in terms of the cosine function.

**Remark 6 .**

The i.v.p.

(83) $$\begin{array}{c} \ddot{x}+n+px+qx^{2}+rx^{3}=0\text{, }x=x\left(t\right), \end{array}$$

given the initial conditions

(84) $$\begin{array}{c} x\left(0\right)=x_{0}\text{ and }x\prime \left(0\right)={\dot{x}}_{0}, \end{array}$$

is a particular case of (75) and (76). Indeed, let *x*(*t*)=*u*(*t*) − (*q*/3*r*). Then, problems (83) and (84) reduce to the problem

(85) $$\begin{array}{c} n+u\left(t\right)\left(p-\frac{q^{2}}{3r}\right)-\frac{pq}{3r}+\frac{2q^{3}}{27r^{2}}+ru{\left(t\right)}^{3}+u^{\prime \prime }\left(t\right)=0, \end{array}$$

(86) $$\begin{array}{c} u\left(0\right)=x_{0}+\frac{q}{3r}\text{ and }u^{\prime }\left(0\right)={\dot{x}}_{0}\text{.} \end{array}$$

**Example 6 .**

Let

(87) $$\begin{array}{c} \ddot{u}+u\left(t\right)+u^{3}\left(t\right)=1\wedge u\left(0\right)=1\wedge u^{\prime }\left(0\right)=1. \end{array}$$

The exact solution is

(88) $$\begin{array}{c} u_{\text{exact}}\left(t\right)=0.29637-\frac{2.45792}{1+℘\left(0.370192-0.709561t;-0.657491,1.34218\right)}, \end{array}$$

with period

(89) $$\begin{array}{c} T_{\text{exact}}=4.24726758. \end{array}$$

An approximate analytical solution is

(90) $$\begin{array}{c} u_{\text{app}}\left(t\right)=1.29637-\frac{2.45792}{0.474364+\left(2.28411/1-\left(3.64963\;\cos\left(0.771764-1.47927t\right)/\sqrt{14.-0.68022\;\cos^{2}\left(0.771764-1.47927t\right)}\right)\right)}, \end{array}$$

with period

(91) $$\begin{array}{c} T_{\text{app}}=4.24748881. \end{array}$$

The error is

(92) $$\begin{array}{c} \max_{-\left(T/2\right)\leq t\leq \left(T/2\right)}\left|u_{\text{exact}}\left(t\right)-u_{\text{app}}\left(t\right)\right|=0.000105178. \end{array}$$

**Figure 6:**
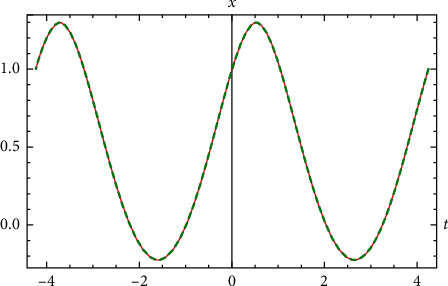
Comparison between the approximate analytical solution and the numerical solution for Example 6.

Comparison between the approximate analytical solution and the numerical solution is shown in Figure 6.

## 5. Damped and Unforced Duffing Equation

Let us consider the i.v.p.

(93) $$\begin{array}{c} \ddot{u}+2\varepsilon \dot{u}+\alpha u+\beta u^{3}=0,\;\varepsilon >\text{0,} \end{array}$$

given that

(94) $$\begin{array}{c} u\left(0\right)=u_{0}\text{ and }u^{\prime }\left(0\right)={\dot{u}}_{0}. \end{array}$$

We will suppose that lim_*t*⟶*∞*_*u*(*t*)=0. Define the residual as

(95) $$\begin{array}{c} R\left(t\right)=u^{\prime \prime }\left(t\right)+2\varepsilon u^{\prime }\left(t\right)+pu\left(t\right)+qu^{3}\left(t\right). \end{array}$$

### 5.1. First Case: *u*_0_ ≠ 0

Assume the ansatz

(96) $$\begin{array}{c} u\left(t\right)=\exp\left(-\rho t\right)\frac{u_{0}\text{cn}\left(f\left(t\right),m\left(t\right)\right)+b_{1}\text{sn}\left(f\left(t\right),m\left(t\right)\right)\text{dn}\left(f\left(t\right),m\left(t\right)\right)}{1+b_{2}{\text{sn}}^{2}\left(f\left(t\right),m\left(t\right)\right)}. \end{array}$$

Then, from results in [22],

(97) $$\begin{array}{c} f^{\prime }{\left(t\right)}^{2}=\alpha -2\varepsilon \rho +\rho^{2}+\beta u_{0}^{2}e^{-2t\rho }\text{ and }m\left(t\right)=\frac{\beta u_{0}^{2}e^{-2t\rho }}{2f^{\prime }{\left(t\right)}^{2}}, \end{array}$$

so that

(98) $$\begin{array}{c} f\left(t\right)=\frac{1}{\rho }\left(\sqrt{\beta u_{0}^{2}+\mu }-\sqrt{\beta u_{0}^{2}e^{-2t\rho }+\mu }+\sqrt{\mu }\left({\text{tanh}}^{-1}\left(\sqrt{1+\frac{\beta u_{0}^{2}e^{-2t\rho }}{\mu }}\right)-{\text{tanh}}^{-1}\left(\sqrt{1+\frac{\beta u_{0}^{2}}{\mu }}\right)\right)\right), \end{array}$$

(99) $$\begin{array}{c} m\left(t\right)=\frac{1/2}{1+\left(\mu e^{2t\rho }/\beta u_{0}^{2}\right)}, \end{array}$$

being

(100) $$\begin{array}{c} \mu =\alpha -2\varepsilon \rho +\rho^{2}. \end{array}$$

The numbers *b*_1_, *b*_2_, and *ρ* are obtained from the following conditions:

(101) $$\begin{array}{c} \left\{\begin{array}{c} u^{\prime }\left(0\right)={\dot{u}}_{0}\text{,}\\ R\left(0\right)=0,\\ R^{\prime }\left(0\right)=0. \end{array}\right. \end{array}$$

Solving the two equations in (88) gives

(102) $$\begin{array}{c} \left\{\begin{array}{c} b_{1}=\frac{u_{0}\rho +{\dot{u}}_{0}}{\sqrt{u_{0}^{2}\beta +\mu }}.\\ b_{2}=\frac{u_{0}^{3}\beta \left(3\alpha -4\varepsilon \rho -3\mu \right)+{\dot{u}}_{0}\left(u_{0}^{2}\beta \left(2\varepsilon -3\rho \right)+2\left(\varepsilon -\rho \right)\left(\alpha +\rho \left(\rho -2\varepsilon \right)\right)\right)+2u_{0}\rho \left(\varepsilon -\rho \right)\left(\alpha +\rho \left(\rho -2\varepsilon \right)\right)}{2u_{0}{\left(u_{0}^{2}\beta +\mu \right)}^{2}} \end{array}\right\}. \end{array}$$

The number *ρ* is a root to the septic

(103) $$\begin{array}{c} -2\varepsilon {\left(u_{0}^{2}\beta +\alpha \right)}^{2}\left(u_{0}^{2}\alpha +u_{0}^{4}\beta +2{\dot{u}}_{0}u_{0}\varepsilon +3{\dot{u}}_{0}^{2}\right)\\ +\left(u_{0}^{2}\beta +\alpha \right)\left(\begin{array}{c} 2u_{0}^{2}\alpha^{2}+5u_{0}^{4}\alpha \beta +8u_{0}^{2}\alpha \varepsilon^{2}-4{\dot{u}}_{0}u_{0}\alpha \varepsilon +6{\dot{u}}_{0}^{2}\alpha +\\ 3u_{0}^{6}\beta^{2}+8u_{0}^{4}\beta \varepsilon^{2}+2{\dot{u}}_{0}u_{0}^{3}\beta \varepsilon +9{\dot{u}}_{0}^{2}u_{0}^{2}\beta +16{\dot{u}}_{0}u_{0}\varepsilon^{3}+24{\dot{u}}_{0}^{2}\varepsilon^{2} \end{array}\right)z\\ -2\left(\begin{array}{c} 8u_{0}^{2}\alpha^{2}\varepsilon -4{\dot{u}}_{0}u_{0}\alpha^{2}+15u_{0}^{4}\alpha \beta \varepsilon -9{\dot{u}}_{0}u_{0}^{3}\alpha \beta +4u_{0}^{2}\alpha \varepsilon^{3}-4{\dot{u}}_{0}u_{0}\alpha \varepsilon^{2}+\\ 18{\dot{u}}_{0}^{2}\alpha \varepsilon +7u_{0}^{6}\beta^{2}\varepsilon -3{\dot{u}}_{0}u_{0}^{5}\beta^{2}+4u_{0}^{4}\beta \varepsilon^{3}+2{\dot{u}}_{0}u_{0}^{3}\beta \varepsilon^{2}+21{\dot{u}}_{0}^{2}u_{0}^{2}\beta \varepsilon +8{\dot{u}}_{0}u_{0}\varepsilon^{4}+12{\dot{u}}_{0}^{2}\varepsilon^{3} \end{array}\right)z^{2}\\ +\left(\begin{array}{c} 8u_{0}^{2}\alpha^{2}+17u_{0}^{4}\alpha \beta +32u_{0}^{2}\alpha \varepsilon^{2}-40{\dot{u}}_{0}u_{0}\alpha \varepsilon +12{\dot{u}}_{0}^{2}\alpha +5u_{0}^{6}\beta^{2}+24u_{0}^{4}\beta \varepsilon^{2}-\\ 38{\dot{u}}_{0}u_{0}^{3}\beta \varepsilon +15{\dot{u}}_{0}^{2}u_{0}^{2}\beta +48{\dot{u}}_{0}^{2}\varepsilon^{2} \end{array}\right)z^{3}\\ -2\left(17u_{0}^{2}\alpha \varepsilon -8{\dot{u}}_{0}u_{0}\alpha +15u_{0}^{4}\beta \varepsilon -9{\dot{u}}_{0}u_{0}^{3}\beta +8u_{0}^{2}\varepsilon^{3}-22{\dot{u}}_{0}u_{0}\varepsilon^{2}+15{\dot{u}}_{0}^{2}\varepsilon \right)z^{4}\\ +2\left(5u_{0}^{2}\alpha +5u_{0}^{4}\beta +16u_{0}^{2}\varepsilon^{2}-18{\dot{u}}_{0}u_{0}\varepsilon +3{\dot{u}}_{0}^{2}\right)z^{5}-4u_{0}\left(5u_{0}\varepsilon -2{\dot{u}}_{0}\right)z^{6}+4u_{0}^{2}z^{7}=0. \end{array}$$

*R*′(0)=0. To avoid solving this, the seventh-degree equation, we may set the default value *ρ*=*ε*. Taking this value for *ρ*, we get the following simplified expressions:

(104) $$\begin{array}{c} b_{1}=\frac{u_{0}\varepsilon +{\dot{u}}_{0}}{\sqrt{u_{0}^{2}\beta +\alpha -\varepsilon^{2}}},\;b_{2}=-\frac{u_{0}\beta \varepsilon \left(u_{0}\varepsilon +{\dot{u}}_{0}\right)}{2{\left(u_{0}^{2}\beta +\alpha -\varepsilon^{2}\right)}^{2}}. \end{array}$$

**Remark 7 .**

In the integrable case, we have *ρ*=2*ε*/3 and then *α*=8/9*ε*^2^ From (99) and (100), *μ*=0 and *m*=1/2. Thus, our approach covers the only integrable case for the damped Duffing equation.

**Example 7 .**

Let

(105) $$\begin{array}{c} \ddot{u}+0.4\dot{u}+u+u^{3}=0\text{,}\\ u\left(0\right)=\frac{1}{4}\text{ and }u^{\prime }\left(0\right)=-\frac{1}{5}. \end{array}$$

The error of the approximate analytical solution compared with numerical solution is

(106) $$\begin{array}{c} \max_{0\leq t\leq 20}\left|u_{\text{app}}\left(t\right)-u_{\text{Ruinge}-\text{Kutta}}\left(t\right)\right|=0.00141579. \end{array}$$

**Figure 7:**
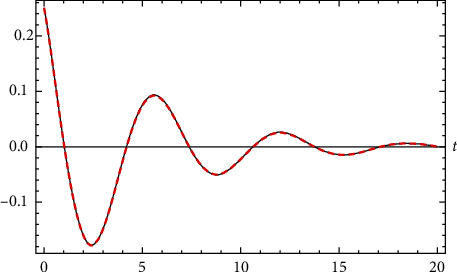
Comparison between the approximate analytical solution and the numerical solution for Example 7.

Comparison between the approximate analytical solution and the numerical solution is shown in Figure 7.

**Example 8 .**

Let

(107) $$\begin{array}{c} \ddot{u}+0.02\dot{u}+u-u^{3}=0\text{,}\\ u\left(0\right)=0.1\text{ and }u^{\prime }\left(0\right)=0. \end{array}$$

The error of the approximate analytical solution compared with numerical solution is

(108) $$\begin{array}{c} \max_{0\leq t\leq 150}\left|u_{\text{app}}\left(t\right)-u_{\text{Ruinge}-\text{Kutta}}\left(t\right)\right|=0.00135922. \end{array}$$

**Figure 8:**
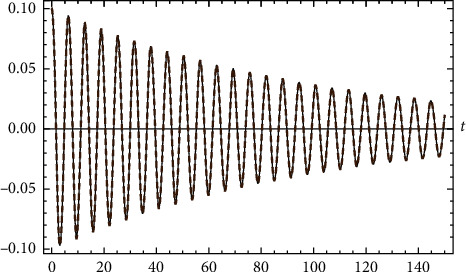
Comparison between the approximate analytical solution and the numerical solution for Example 8.

Comparison between the approximate analytical solution and the numerical solution is shown in Figure 8.

**Remark 8 .**

Let *ρ*=*ε*. An approximate analytical solution to the i.v.p.

(109) $$\begin{array}{c} \ddot{u}+2\varepsilon \dot{u}+\alpha u+\beta u^{3}=0\text{,}\\ u\left(0\right)=u_{0}\text{ and }u^{\prime }\left(0\right)=0, \end{array}$$

is given by

(110) $$\begin{array}{c} u\left(t\right)=\frac{u_{0}}{1-\left(u_{0}^{2}\beta \varepsilon^{2}/2{\left(u_{0}^{2}\beta +\alpha -\varepsilon^{2}\right)}^{2}\right)sn{\left(f\left(t\right)|m\left(t\right)\right)}^{2}}\left(cn\left(f\left(t\right)|m\left(t\right)\right)+\frac{\varepsilon }{\sqrt{u_{0}^{2}\beta +\alpha -\varepsilon^{2}}}dn\left(f\left(t\right)|m\left(t\right)\right)sn\left(f\left(t\right)|m\left(t\right)\right)\right)e^{-t\varepsilon }, \end{array}$$

where *f*(*t*) and *m*(*t*) are given by (98) and (99).

### 5.2. Second Case: *u*_0_=0

Let

(111) $$\begin{array}{c} \ddot{u}+2\varepsilon \dot{u}+\alpha u+\beta u^{3}=0\text{,}u\left(0\right)=0\text{ and }u^{\prime }\left(0\right)={\dot{u}}_{0}\text{.} \end{array}$$

Assuming the ansatz [22],

(112) $$\begin{array}{c} u\left(t\right)=-\lambda \sqrt{1-m_{0}\left(t\right)}\exp\left(-\rho t\right)s\;d\left(f_{0}\left(t\right),m_{0}\left(t\right)\right), \end{array}$$

we will have

(113) $$\begin{array}{c} f_{0}^{\prime }{\left(t\right)}^{2}=\frac{1}{2}\beta \lambda^{2}e^{-2t\rho }+\kappa , \end{array}$$

(114) $$\begin{array}{c} m_{0}\left(t\right)=-\frac{\beta \lambda^{2}}{2f_{0}^{\prime }{\left(t\right)}^{2}}e^{-2t\rho }. \end{array}$$

Then,

(115) $$\begin{array}{c} f\left(t\right)=\frac{1}{2\rho }\left(\begin{array}{c} \sqrt{4\kappa +2\beta \lambda^{2}}-\sqrt{4\kappa +2\beta \lambda^{2}e^{-2t\rho }}+\\ 2\sqrt{\kappa }\tanh^{-1}\left(\sqrt{1+\frac{q\lambda^{2}}{2\kappa }e^{-2t\rho }}\right)-2\sqrt{\kappa }\tanh^{-1}\left(\sqrt{1+\frac{q\lambda^{2}}{2\kappa }}\right) \end{array}\right), \end{array}$$

(116) $$\begin{array}{c} m\left(t\right)=-\frac{1}{1+\left(2\kappa /\beta \lambda^{2}\right)e^{2t\rho }}\text{, }\kappa =p-2\rho \varepsilon +\rho^{2}. \end{array}$$

The number *λ* is found form the initial condition $u^{\prime }\left(0\right)={\dot{u}}_{0}$, and its value reads

(117) $$\begin{array}{c} \lambda =-\frac{\sqrt{\left(\sqrt{4{\dot{x}}_{0}^{2}\beta +{\left(\alpha -2\varepsilon \rho +\rho^{2}\right)}^{2}}-\alpha +2\varepsilon \rho -\rho^{2}/\beta \right)}}{\sqrt{2}}. \end{array}$$

The number *ρ* is a solution to some decic equation. Default value is *ρ*=*ε*.

**Example 9 .**

Let

(118) $$\begin{array}{c} \ddot{u}+0.1\dot{u}+u+u^{3}=0\text{,}\;u\left(0\right)=0\text{ and }u^{\prime }\left(0\right)=-0.1. \end{array}$$

The error of the approximate analytical solution compared with numerical solution is

(119) $$\begin{array}{c} \max_{0\leq t\leq 150}\left|u_{\text{app}}\left(t\right)-u_{\text{Ruinge}-\text{Kutta}}\left(t\right)\right|=0.000303296. \end{array}$$

**Figure 9:**
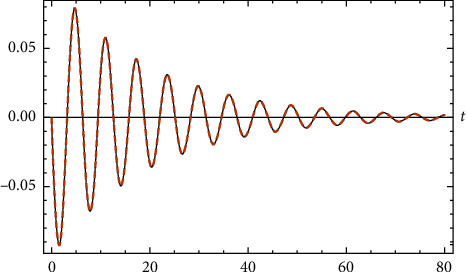
Comparison between the approximate analytical solution and the numerical solution for Example 9.

Comparison between the approximate analytical solution and the numerical solution is shown in Figure 9.

## 6. Damped and Forced Duffing Equation

Let us consider the Duffing equation as originally was introduced by Georg Duffing:

(120) $$\begin{array}{c} \ddot{x}+r\dot{x}+\omega_{0}^{2}x+\beta x^{3}=F\;\cos\;\omega t, \end{array}$$

given the initial conditions

(121) $$\begin{array}{c} x\left(0\right)=x_{0}\text{ and }x^{\prime }\left(0\right)={\dot{x}}_{0}. \end{array}$$

Let

(122) $$\begin{array}{c} x\left(t\right)=u\left(t\right)+c_{1}\cos\;\omega t+c_{2}\sin\;\omega t. \end{array}$$

We will suppose that the function *u*=*u*(*t*) is a solution to the Duffing equation

(123) $$\begin{array}{c} \ddot{u}+r\;\dot{u}+\alpha u+\beta u^{3}=0\text{,}\\ u\left(0\right)=x_{0}-c_{1}\text{ and }u^{\prime }\left(0\right)={\dot{x}}_{0}-c_{2}\omega , \end{array}$$

where

(124) $$\begin{array}{c} \alpha =\frac{1}{2}\left(3\;c_{1}^{2}\;\beta +3\;c_{2}^{2}\;\beta +2\;\omega_{0}^{2}\right). \end{array}$$

The numbers *c*_1_ and *c*_2_ are chosen, so that

(125) $$\begin{array}{c} 432F^{2}\beta^{3}c_{1}^{3}+1152Fr^{2}\beta^{2}\omega^{2}c_{1}^{2}-192\beta \left(3F^{2}\beta \omega^{2}-3F^{2}\beta \omega_{0}^{2}-4r^{4}\omega^{4}-4r^{2}\omega^{6}+8r^{2}\omega_{0}^{2}\omega^{4}-4r^{2}\omega_{0}^{4}\omega^{2}\right)c_{1}-192F\beta \left(3F^{2}\beta -4r^{2}\omega^{4}+4r^{2}\omega_{0}^{2}\omega^{2}\right)=0 \end{array}$$

(126) $$\begin{array}{c} 432F^{2}\beta^{3}c_{2}^{3}-1152c_{2}^{2}Fr\beta^{2}\omega \left(\omega^{2}-\omega_{0}^{2}\right)c_{2}^{2}+768r^{2}\beta \omega^{2}\left(r^{2}\omega^{2}+\omega^{4}-2\omega_{0}^{2}\omega^{2}+\omega_{0}^{4}\right)c_{2}-768Fr^{3}\beta \omega^{3}=0. \end{array}$$

**Example 10 .**

Let

(127) $$\begin{array}{c} \ddot{x}+0.1\dot{x}+x+x^{3}=0.1\;\cos\;0.4\;t,x\left(0\right)=0\text{ and }x^{\prime }\left(0\right)=0. \end{array}$$

The error of the approximate analytical solution compared with numerical solution is

(128) $$\begin{array}{c} \max_{0\leq t\leq 150}\left|x_{\text{app}}\left(t\right)-x_{\text{Ruinge}-\text{Kutta}}\left(t\right)\right|=0.00287382. \end{array}$$

The approximate solution is

(129) $$\begin{array}{c} x\left(t\right)=e^{-0.05t}\frac{\left(-0.117339\text{cn}-0.00791022\text{dnsn}\right)}{1+1.411\times 10^{-7}{\text{sn}}^{2}}+0.00551957\;\sin\left(0.4t\right)+0.117339\;\cos\left(0.4t\right), \end{array}$$

where

(130) $$\begin{array}{c} cn=cn\left(f\left(t\right),m\left(t\right)\right),\;sn=sn\left(f\left(t\right),m\left(t\right)\right),\;dn=dn\left(f\left(t\right),m\left(t\right)\right),\\ f\left(t\right)=-20.1343\sqrt{1.0182+0.0137685e^{-0.0993331t}}\\ -5.07916\;\log\left({\left(1-0.991023\sqrt{1.0182+0.0137685e^{-0.0993331t}}\right)}^{2}\right)\\ +10.1583\;\log\left(0.991023\sqrt{1.0182+0.0137685e^{-0.0993331t}}+1\right)-37.4127,\\ m\left(t\right)=\frac{1}{147.903e^{0.0993331t}+2}. \end{array}$$

**Figure 10:**
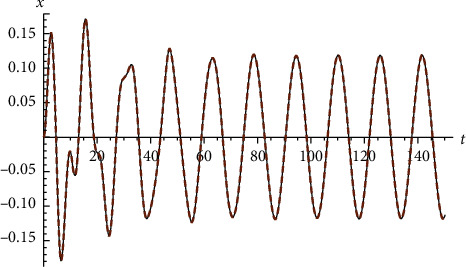
Comparison between the approximate analytical solution and the numerical solution for Example 10.

Comparison between the approximate analytical solution and the numerical solution is shown in Figure 10.

Finally, let us compare the accuracy of the obtained results in comparation with the homotopy perturbation method (HPM). This method gives the approximate solution:

(131) $$\begin{array}{c} x_{\text{HPM}}\left(t\right)=\frac{e^{-\left(1/2\right)t\left(r+\kappa \right)}}{2\kappa^{2}\left(r^{2}\omega^{2}+{\left(\omega^{2}-\omega_{0}^{2}\right)}^{2}\right)}, \end{array}$$

(132) $$\begin{array}{c} \left(F\left(r^{2}\left(\omega^{2}-\omega_{0}^{2}\right)\left(e^{t\kappa }+1\right)-r\kappa \left(\omega^{2}+\omega_{0}^{2}\right)\left(e^{t\kappa }-1\right)+4\omega_{0}^{2}\left(\omega_{0}^{2}-\omega^{2}\right)\left(e^{t\kappa }+1\right)\right)+2F\kappa^{2}e^{\frac{1}{2}t\left(r+\kappa \right)}\left(r\omega \;\sin\left(t\omega \right)+\left(\omega_{0}^{2}-\omega^{2}\right)\cos\left(t\omega \right)\right)+\left(r^{2}\omega^{2}+{\left(\omega^{2}-\omega_{0}^{2}\right)}^{2}\right)\left(x_{0}\left(r\left(\left(r+\kappa \right)e^{t\kappa }+r-\kappa \right)-4\omega_{0}^{2}\left(e^{t\kappa }+1\right)\right)+2{\dot{x}}_{0}\kappa \left(e^{t\kappa }-1\right)\right)\right), \end{array}$$

(133) $$\begin{array}{c} \kappa =\sqrt{r^{2}-4\omega_{0}^{2}}. \end{array}$$

The error of this approximation compared with numerical solution is

(134) $$\begin{array}{c} {\text{Max}}_{0\leq t\leq 150}\left|x_{\text{HPM}}\left(t\right)-x_{Ruinge-Kutta}\left(t\right)\right|=0.0136613. \end{array}$$

**Figure 11:**
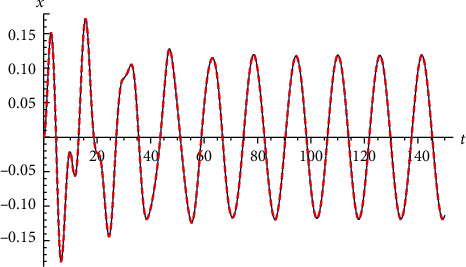
Comparison between the approximate analytical solution and the homotopy solution for Example 10.

Comparison between the approximate analytical solution and the homotopy solution is shown in Figure 11.

## 7. Analysis and Discussion

We have solved the undamped and constantly forced Duffing equation exactly. Trigonometric approximant was also provided. For the damped or forced case, we derived approximate analytical solution. As far as we know, the Duffing equation (1) has not been solved using the tools we employed in this work. For the damped unforced case, author in [8] obtained approximate analytical solution using generalized Jacobian elliptic functions. More exactly, author considered the following equation:

(135) $$\begin{array}{c} \ddot{x}+2\beta \dot{x}+\alpha x-\varepsilon x^{3}=0\text{, }x\left(0\right)=x_{0}\text{ and }x^{\prime }\left(0\right)={\dot{x}}_{0}. \end{array}$$

The obtained solution in [8] has the form

(136) $$\begin{array}{c} x\left(t\right)=c_{0}\exp\left(-\beta t\right)sn\left(\omega \left(t\right)+c_{1},m\left(t\right)\right), \end{array}$$

where

(137) $$\begin{array}{c} \omega \left(t\right)=\frac{1}{2\beta }\left(\sqrt{2}\left(\sqrt{2\alpha -2\beta^{2}-c_{0}^{2}\varepsilon }-\sqrt{2\alpha -2\beta^{2}-c_{0}^{2}\varepsilon e^{-2\beta t}}\right)+2c_{0}\sqrt{\varepsilon }\sqrt{\beta^{2}-\alpha }\left(\frac{\sqrt{1-\left(2\left(\alpha -\beta^{2}\right)/c_{0}^{2}\varepsilon \right)}{\text{csch}}^{-1}\left(c_{0}\sqrt{\varepsilon }/\sqrt{2\beta^{2}-2\alpha }\right)}{\sqrt{2\alpha -2\beta^{2}-c_{0}^{2}\varepsilon }}+\frac{e^{\beta t}\sqrt{2\alpha -2\beta^{2}-c_{0}^{2}\varepsilon e^{-2\beta t}}{\text{csch}}^{-1}\left(c_{0}\sqrt{\varepsilon }e^{\beta \left(-t\right)}/\sqrt{2\beta^{2}-2\alpha }\right)}{c_{0}^{2}\varepsilon \sqrt{\left(2\left(\beta^{2}-\alpha \right)e^{2\beta t}/c_{0}^{2}\varepsilon \right)+1}}\right)\right), \end{array}$$

(138) $$\begin{array}{c} m\left(t\right)=-\frac{c_{0}^{2}\varepsilon }{c_{0}^{2}\varepsilon +2\left(\beta^{2}-\alpha \right)e^{2\beta t}}. \end{array}$$

The constants *c*_0_ and *c*_1_ are determined from the initial conditions as follows:

(139) $$\begin{array}{c} c_{1}=sn^{-1}\left(x_{0}/c_{0},m\left(0\right)\right), \end{array}$$

(140) $$\begin{array}{c} c_{0}=\pm \sqrt{\frac{\alpha }{\varepsilon }-\frac{\beta^{2}}{\varepsilon }\pm \frac{\sqrt{\alpha^{2}-2\alpha \beta^{2}+\beta^{4}-2\alpha \varepsilon x_{0}^{2}-4\beta \varepsilon x_{0}{\dot{x}}_{0}+\varepsilon^{2}x_{0}^{4}-2\varepsilon {\dot{x}}_{0}^{2}}}{\varepsilon }}. \end{array}$$

This approach is different from the method we used in this work. Let us compare the solution (136) with the solution we obtained in Example 8:

(141) $$\begin{array}{c} \ddot{u}+0.02\dot{u}+u-u^{3}=0\text{,}u\left(0\right)=0.1\text{ and }u^{\prime }\left(0\right)=0\text{ for }0\leq t\leq 150. \end{array}$$

Using formula gives the approximate analytical solution

(142) $$\begin{array}{c} x\left(t\right)=0.100005e^{-0.01t}sn\left(50.\left(\sqrt{2}\left(1.41767-\sqrt{1.9998+0.010001e^{-0.02t}}\right)-0.2\left(33.4329-\frac{99.9901e^{0.01t}\sqrt{1.9998+0.010001e^{-0.02t}}\csc\;h^{-1}\left(0.0707177e^{-0.01t}\right)}{\sqrt{1+199.96e^{0.02t}}}\right)\right)+1.55892,\frac{0.010001}{-0.010001-1.9998e^{0.02t}}\right). \end{array}$$

The error of this solution compared with the Runge–Kutta numerical method equals 0.00142148. The error obtained in our method equals 0.00135922. We may try a simpler ansatz in the form

(143) $$\begin{array}{c} x\left(t\right)=c_{0}\exp\left(-\rho t\right)\sin\left(w\left(t\right)\right), \end{array}$$

with

(144) $$\begin{array}{c} w\left(t\right)=\frac{\begin{array}{c} -\sqrt{4\alpha +4\rho \left(\rho -2\beta \right)-3c_{0}^{2}\varepsilon }+\sqrt{4\alpha +4\rho \left(\rho -2\beta \right)-3c_{0}^{2}\varepsilon e^{-2\rho t}}+-\sqrt{4\alpha +4\rho \left(\rho -2\beta \right)-3c_{0}^{2}\varepsilon }+\sqrt{4\alpha +4\rho \left(\rho -2\beta \right)-3c_{0}^{2}\varepsilon e^{-2\rho t}}+2\sqrt{\alpha +\rho \left(\rho -2\beta \right)}\\ \left({\text{coth}}^{-1}\left(\left(2\sqrt{\alpha +\rho \left(\rho -2\beta \right)}/\sqrt{4\alpha +4\rho \left(\rho -2\beta \right)-3c_{0}^{2}\varepsilon }\right)\right)-{\text{coth}}^{-1}\left(\left(2\sqrt{\alpha +\rho \left(\rho -2\beta \right)}/\sqrt{4\alpha +4\rho \left(\rho -2\beta \right)-3c_{0}^{2}\varepsilon e^{-2\rho t}}\right)\right)\right) \end{array}}{2\rho }. \end{array}$$

The numbers *c*_0_ and *ρ* are determined from the system

(145) $$\begin{array}{c} -8\alpha \beta +8\alpha \rho +16\beta^{2}\rho -24\beta \rho^{2}+6\beta c_{0}^{2}\varepsilon -9c_{0}^{2}\varepsilon \rho +8\rho^{3}=0,\\ -4\alpha c_{0}^{2}+8\beta c_{0}^{2}\rho +3\beta c_{0}^{4}\varepsilon -4c_{0}^{2}\rho^{2}-3c_{0}^{2}\varepsilon x_{0}^{2}+4\alpha x_{0}^{2}+8\beta \rho x_{0}^{2}+8\rho^{2}x_{0}^{2}+8\rho x_{0}{\dot{x}}_{o}+4{\dot{x}}_{0}^{2}=0. \end{array}$$

Using this ansatz, we obtain the approximate analytical solution:

(146) $$\begin{array}{c} x_{\text{trigo}}\left(t\right)=0.100005e^{-0.00996291t}\\ \sin\left(1.56087+50.1861\left(\sqrt{3.9996+0.030003e^{-0.0199258t}}+1.9999\left(-{\text{coth}}^{-1}\left(\frac{1.9999}{\sqrt{3.9996+0.030003e^{-0.0199258t}}}\right)+\left(3.14134-1.5708i\right)\right)-2.00739\right)\right) \end{array}$$

The error of the trigonometric solution (146) compared with the numerical solution using the Runge–Kutta method equals 0.00195254, so that the trigonometric solution is good as well.

## 8. Conclusions and Future Work

The methods employed here may be useful to study other nonlinear oscillators of the form

(147) $$\begin{array}{c} \ddot{x}+2\delta \;\dot{x}+f\left(x\right)=F\;\cos\;\omega t,x\left(0\right)=x_{0}\text{ and }x^{\prime }\left(0\right)={\dot{x}}_{0}, \end{array}$$

where the function *f* is odd: *f*(−*x*)=−*f*(*x*). To this end, we approximate this function on some interval [−*A*, *A*] by means of Chebyshev polynomials in the form

(148) $$\begin{array}{c} f\left(x\right)\approx px+qx^{3}. \end{array}$$

Then, the i.v.p. is replaced with the i.v.p.

(149) $$\begin{array}{c} \ddot{x}+2\delta \;\dot{x}+px+qx^{3}=F\;\cos\;\omega t,\;x\left(0\right)=x_{0}\text{ and }x^{\prime }\left(0\right)={\dot{x}}_{0}. \end{array}$$

On the other hand, we may study the following cubic quintic Duffing oscillator:

(150) $$\begin{array}{c} \ddot{x}+2\delta \;\dot{x}+px+qx^{3}+rx^{5}=F\;\cos\;\omega t,x\left(0\right)=x_{0}\text{ and }x^{\prime }\left(0\right)={\dot{x}}_{0}. \end{array}$$

For the unforced and damped cases, we may try the ansatz

(151) $$\begin{array}{c} x\left(t\right)=c_{0}\exp\left(-\rho t\right)\sin\left(f\left(t\right)+\text{arccos}\left(\frac{x_{0}}{c_{0}}\right)\right), \end{array}$$

where

(152) $$\begin{array}{c} f\left(t\right)=\frac{1}{2\sqrt{2}}\int_{0}^{t}\sqrt{8p-16\delta \rho +8\rho^{2}+6c_{0}^{2}e^{-2\tau \rho }q+5c_{0}^{4}e^{-4\tau \rho }r}\text{d}\tau , \end{array}$$

and

(153) $$\begin{array}{c} 8\left(px_{0}^{2}-2\delta \rho x_{0}^{2}+2\rho^{2}x_{0}^{2}+2\rho {\dot{x}}_{0}x_{0}+{\dot{x}}_{0}^{2}\right)-2\left(-8\delta \rho +4p+4\rho^{2}-3qx_{0}^{2}\right)c_{0}^{2}+\left(5rx_{0}^{2}-6q\right)c_{0}^{4}-5rc_{0}^{6}=0. \end{array}$$

The number *ρ* is a free parameter that is chosen in order to minimize the residual error

## Figures and Tables

**Table 1:** Errors for approximations (46) and (47).

*m*	Max_−*T*≤*t*≤*T*_|*sn*(*t*, *m*) − sin_*m*_(*t*)|	Max_−*T*≤*t*≤*T*_|*cn*(*t*, *m*) − cos_*m*_(*t*)|
0	0	0
0.05	0.0000186142	0.0000332484
0.1	0.0000775641	0.000139051
0.15	0.000182102	0.000327722
0.2	0.000338416	0.000611534
0.25	0.000553861	0.00100522
0.3	0.000837256	0.00152665
0.35	0.0011993	0.00219775
0.4	0.00165312	0.00304575
0.45	0.00221506	0.00410502
0.5	0.00290582	0.00541969
0.55	0.00375209	0.00704758
0.6	0.00478916	0.0.0090664
0.66	0.00606499	0.0115838
0.7	0.00764718	0.0147546
0.75	0.00963546	0.0188129
0.8	0.0121865	0.0241364
0.85	0.055962	0.0313949
0.9	0.0203137	0.0419635
0.95	0.0277681	0.0595356

**Table 2:** Errors for approximations (52) and (53).

*m*	Max_−*T*≤*t*≤*T*_|*sn*(*t*, *m*) − sin_*m*_(*t*)|	Max_−*T*≤*t*≤*T*_|*cn*(*t*, *m*) − cos_*m*_(*t*)|
0	0	0
0.05	8.066*e*-9	6.548*e*-9
0.1	1.4551*e*-7	1.158*e*-7
0.15	8035*e*-7	6.51*e*-7
0.2	3.0125*e*-6	2.23*e*-6
0.25	8.45*e*-6	6.29*e*-6
0.3	0.00002	0.000015
0.35	0.00004	0.000031
0.4	0.000089	0.000061
0.45	0.00017	0.00011
0.5	0.00032	0.00021
0.55	0.00058	0.00037
0.6	0.0011	0.00065
0.65	0.0019	0.0012
0.7	0.0036	0.0021
0.75	0.0071	0.0039
0.8	0.0146	0.0079
0.85	0.0327	0.0173
0.9	0.0832	0.043
0.95	0.265	0.136

## Data Availability

No data were used to support this study.
